# Nuclear cGAS: sequestration and beyond

**DOI:** 10.1007/s13238-021-00869-0

**Published:** 2021-08-09

**Authors:** Juli Bai, Feng Liu

**Affiliations:** 1 Departments of Pharmacology, University of Texas Health at San Antonio, San Antonio, TX USA; 2 National Clinical Research Center for Metabolic Diseases, Metabolic Syndrome Research Center, Key Laboratory of Diabetes Immunology, Ministry of Education, and Department of Metabolism and Endocrinology, The Second Xiangya Hospital of Central South University, Changsha, 410011 China

**Keywords:** cGAS, STING, innate immunity, nuclear translocation, DNA damage repair, micronuclei

## Abstract

The cyclic GMP-AMP (cGAMP) synthase (cGAS) has been identified as a cytosolic double stranded DNA sensor that plays a pivotal role in the type I interferon and inflammation responses via the STING-dependent signaling pathway. In the past several years, a growing body of evidence has revealed that cGAS is also localized in the nucleus where it is associated with distinct nuclear substructures such as nucleosomes, DNA replication forks, the double-stranded breaks, and centromeres, suggesting that cGAS may have other functions in addition to its role in DNA sensing. However, while the innate immune function of cGAS is well established, the non-canonical nuclear function of cGAS remains poorly understood. Here, we review our current understanding of the complex nature of nuclear cGAS and point to open questions on the novel roles and the mechanisms of action of this protein as a key regulator of cell nuclear function, beyond its well-established role in dsDNA sensing and innate immune response.

## INTRODUCTION

**Figure 1:**
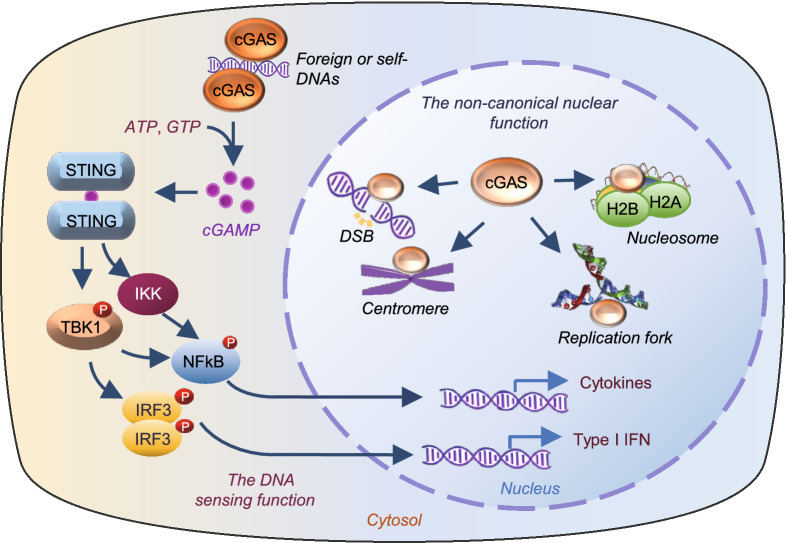
**Canonical role of cytosolic cGAS and non-canonical role of nuclear cGAS.** In the cytosol, cGAS is activated by self- or non-self dsDNAs, leading to dimerization-induced activation and cGAMP production, which in turn promotes IFN gene expression by activating the STING-TBK1-IRF3 axis. The binding of cGAMP to STING also leads to the activation of the IKK/NFκB pathway, resulting in inflammatory gene expression. In the nucleus, cGAS is localized at DSB, the replication fork, centromere, and/or nucleosome where it carries out the nonconical roles beyond innate immune function

The innate immune system recognizes microorganisms and provides the first line of host defense against pathogens. In vertebrates, pathogen-derived double stranded DNA (dsDNA) is sensed in the cytosol by cyclic GMP-AMP (cGAMP) synthase (cGAS, also known as MB21D1), which catalyzes the synthesis of a cyclic dinucleotide cGAMP (Sun et al., 2013; Wu et al., 2013). The second message cGAMP binds to the signaling adaptor protein STING (stimulator of interferon genes), leading to activation of TANK-binding kinase 1 (TBK1) and consequently phosphorylation of interferon regulatory factor 3 (IRF3). STING activation also increases the phosphorylation and activation of nuclear factor-kB (NF-κB) via TBK1 or IkB kinaseε (IKKε) (Abe and Barber, 2014; Fang et al., 2017; Balka et al., 2020). Phosphorylation promotes the nuclear translocation of IRF3 and NFκB, leading to the expression of type I interferon (IFN) and cytokine genes (Fig. 1). In addition to playing a pivotal role in innate immune defense against invading pathogens, cGAS also recognizes host DNAs such as mitochondrial DNA (mtDNA) aberrantly localized in the cytosol (West et al., 2015), contributing to increased metabolic disorders such as sterile inflammation, insulin resistance, and the development of nonalcoholic fatty liver disease (NAFLD) (Bai et al., 2017, 2020; Bai and Liu, 2019). cGAS can also be activated by cytosolic localized DNA abnormally released from the nucleus after cell damage or from broken or dead cells after phagocytosis (Chen et al., 2016; Gluck et al., 2017; Harding et al., 2017; Yang et al., 2017), or by DNA in micronuclei results from chromosome segregation errors (Janssen et al., 2011; Harding et al., 2017), providing a surveilling mechanism linking genome instability and/or DNA damage-induced cellular dysfunctions to innate immunity.

cGAS was first proposed as cytosolic DNA sensor in 2006 (Sun et al., 2013), thereby, compartmentalization of self-DNA in mitochondria and nucleus is thought to be essential for cGAS to discriminate non-self- versus self-DNA (Sun et al., 2013). However, many recent studies show that cGAS is constitutively, and perhaps even predominantly in the nucleus (Orzalli et al., 2015; Mackenzie et al., 2017; Lahaye et al., 2018; Liu et al., 2018; Gentili et al., 2019; Jiang et al., 2019; Chen et al., 2020; Cui et al., 2020). In the nucleus, cGAS is tethered tightly by a salt-resistant interaction with intact chromatin (Volkman et al., 2019), and this molecule could interact with a variety of nuclear substructures such as nucleosomes (Zierhut et al., 2019; Michalski et al., 2020; Pathare et al., 2020; Zhao et al., 2020), the double-stranded breaks (DSBs) (Liu et al., 2018), centromeres and LINE DNA repeats (Gentili et al., 2019), and DNA replication forks (Chen et al., 2020), suggesting a potential broad function of the nuclear localized cGAS (Fig. 1). The nuclear tethered cGAS is either inactive or with a very low activity compared to the cytosolic localized cGAS, suggesting the presence of a mechanism that prevents cGAS, a member of the cGAS/DncV-like nucleotidyltransferase (CD-NTase) family that recognizes DNA via a sequence-independent manner, from activation by self-DNA. Here, we review the complex nature of the nuclear localized cGAS, discuss models that prevent cGAS activation by nuclear DNA, and point to current limitations and open questions in understanding the regulation and function of nuclear cGAS.

## THE STRUCTURE OF cGAS

**Figure 2:**
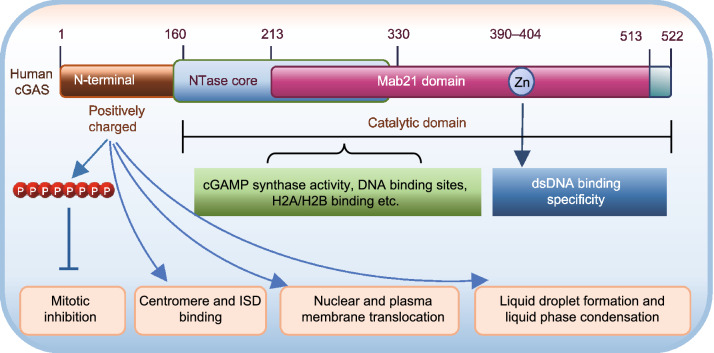
**Sequence Structure and Structure-based Function of cGAS.** Human cGAS consists of a ~160 amino acid long unstructured and positively charged N terminus and a 360 amino acid C-terminal fragment that contains a nucleotidyltransferase (NTase) core domain (160–330) and the male abnormal 21 (Mab21) domain (213–513). The NTase domain is critical for the enzyme activity of cGAS. The Mab21 domain contains a zinc-ribbon structural domain (390–404) that is important to scale the specificity of cGAS toward dsDNA. Hyperphosphorylated N-terminus of cGAS is critical in suppressing cGAS activity during mitosis. This less evolutionarily conserved sequence also plays a key role in determining nuclear, cytoplasmic distribution, sensing nuclear chromatin, binding to immune stimulatory DNA (ISD), plasma membrane, or centromere as well as assembly of lipid phase condensation

**Figure 3:**
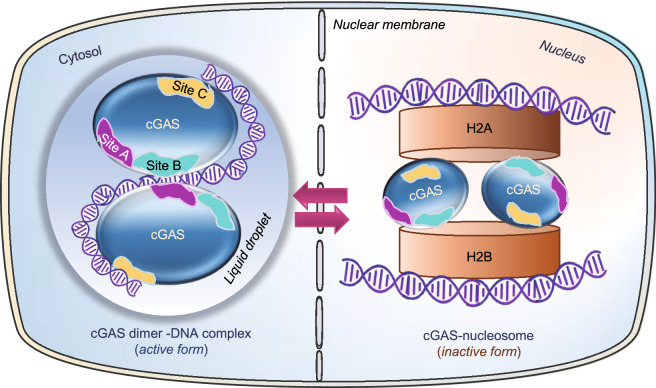
**Schematics of cGAS in cytoplasmic active state and in nuclear inactive state.** In the cytosol, cGAS interacts with dsDNA via DNA binding site A (purple) or B (teal) or C (yellow), which leads to the formation of cGAS dimerization and DNA liquid phase condensation, thus activating its enzymatic function. In the nucleus, cGAS site B interacts with the histones H2A-H2B of the nucleosome, which blocks cGAS dimerization and thus activation

Human cGAS consists of a ~160 amino acid long unstructured and positively charged N-terminus and a 360 amino acid C-terminal fragment that contains a NTase core domain (160–330) and the male abnormal 21 (Mab21) domain (213–513) (Wu et al., 2014). The NTase domain contains several conserved amino acid residues within the NTase superfamily including G^212^, S^213^, E^225^, D^227^ and D^319^ that are critical for the enzyme activity of cGAS. The Mab21 domain contains a zinc-ribbon structural domain (390–404) typically defined as H(X5)CC(X6)C, which has been shown to be functionally important to scale the specificity of cGAS toward dsDNA (Wu et al., 2014; Kranzusch, 2019) (Fig. 2). While the N-terminus of cGAS is less well characterized structurally and less evolutionarily conserved, many recent studies show that this disordered region plays critical roles in sensing nuclear chromatin (Li et al., 2021), binding to immune stimulatory DNA (ISD) (Sun et al., 2013), interacting with phosphatidylinositol 4,5-bisphosphate (PI(4,5)P2) to localize cGAS at the plasma membrane (Barnett et al., 2019), facilitating nuclear cGAS positioning for centromere binding and innate immune activation (Gentili et al., 2019), as well as promoting efficient DNA-induced assembly of lipid phase condensation (Du and Chen, 2018). Hyperphosphorylation at the N-terminus greatly inhibits cGAS activity during mitosis (Li et al., 2021). Human cGAS has A, B and C three DNA binding sites composed of positively charged key DNA-interactive amino acid residues (Civril et al., 2013; Li et al., 2013; Zhang et al., 2014; Zhou et al., 2018; Xie et al., 2019) (Fig. 3). Human and mouse cGAS proteins share <60% amino acid identity and there are 116 amino acid differences in NTase domain between human and mouse cGAS proteins (Zhou et al., 2018), which causes a great reduction in human cGAS enzymatic activity compared to other mammalian cGAS homologs (Zhou et al., 2018). cGAS is activated by double-stranded DNA (dsDNA) in a sequence-independent (Civril et al., 2013) but length-dependent manner and activation of cGAS occurs more efficiently with longer DNA (Andreeva et al., 2017; Luecke et al., 2017). The binding of DNA ligands to sites A and B induces cGAS dimerization, with two DNA strands sandwiched between the two cGAS monomers (Li et al., 2013; Zhang et al., 2014) (Fig. 3). Dimerization switches cGAS from the catalytic inactive state to the catalytic active state, which is further promoted by clustering along longer DNA (Andreeva et al., 2017; Hooy and Sohn, 2018). The site C DNA interface of cGAS works together with the N-terminal domain to facilitate liquid-phase condensation and consequent cGAS activation (Xie et al., 2019) (Fig. 3).

### Structure-based sequestration and inhibition of nuclear cGAS by nucleosomes

In the nucleus of eukaryotic cells, the long DNA molecules wraps around histone proteins such as histone 2A (H2A) and histone 2B (H2B) to form nucleosomes, which are further packed into a more compact denser structure, the chromatin (Luger et al., 2012). Several recent cryoelectronic microscopic structure and functional studies found that cGAS binds with a higher affinity to nucleosomes compared to naked DNA, which greatly blocks cGAS dimerization and activity (Zierhut et al., 2019; Boyer et al., 2020; Kujirai et al., 2020; Michalski et al., 2020; Pathare et al., 2020; Wang et al., 2020; Zhao et al., 2020). Although the naked dsDNA in the linker region between nucleosomes can still activate cGAS, most of the nucleosome-bound dsDNA does not activate cGAS efficiently (Lahaye et al., 2018; Zierhut et al., 2019). The binding of two human cGAS monomers to two nucleosome core particles (NPCs) forms a sandwich-like structure (Kujirai et al., 2020; Pathare et al., 2020), which is mediated by the interaction between second DNA binding site (Site B) with a negatively charged acidic patch formed by H2A and 2B in the nucleosomes (Boyer et al., 2020; Cao et al., 2020; Kujirai et al., 2020; Michalski et al., 2020; Pathare et al., 2020; Zhao et al., 2020) rather than nucleosomal DNA (Fig. 3). The binding of the catalytic domain of cGAS to the acidic patch of H2A-H2B in a nucleosome buries the DNA binding site B and thus locks cGAS into a monomeric state, which prevents cGAS dimerization and thus activation (Volkman et al., 2019; Zierhut et al., 2019; Kujirai et al., 2020; Michalski et al., 2020; Pathare et al., 2020; Zhao et al., 2020). Although site A is solvent-exposed in the cGAS-nucleosome core particle (NCP) complex, its mutations do not affect the affinity of cGAS for mono-nucleosomes nor the association of cGAS with mitotic chromosomes (Kujirai et al., 2020). Site C residues within the α-Helix or KRKR loop of human cGAS may interact with nucleosomal DNA around the superhelical location 3 or 4 (SHL3 or SHL4) and mutations at these sites led to the formation of complexes with discrete sizes rather than large multimers, indicating that site C is required for human cGAS to generate NCP stacks, but not mouse cGAS due to a less conserved sequence (Kujirai et al., 2020; Pathare et al., 2020) (Fig. 3). Disrupting the interaction of cGAS with the acidic patch of nucleosomes is in itself sufficient to trigger innate immune activation (Cao et al., 2020; Pathare et al., 2020). A recent study showed that mutations in the *LSM11* (U7 snRNA-associated Sm-like protein) and *RNU7-1* (RNA, U7 small nuclear 1) genes, which encode core components of histone mRNA-preprocessing complex, lead to the accumulation of misprocessed linker-histone mRNAs and altered histone stoichiometry, resulting in cGAS disassociation from nucleosome and activation of IFN response (Uggenti et al., 2020). These findings revealed a mechanism by which nuclear cGAS is sequestered by chromatin to optimize the dynamic regulation of the immune responses.

## THE NUCLEAR FUNCTION OF cGAS

**Figure 4:**
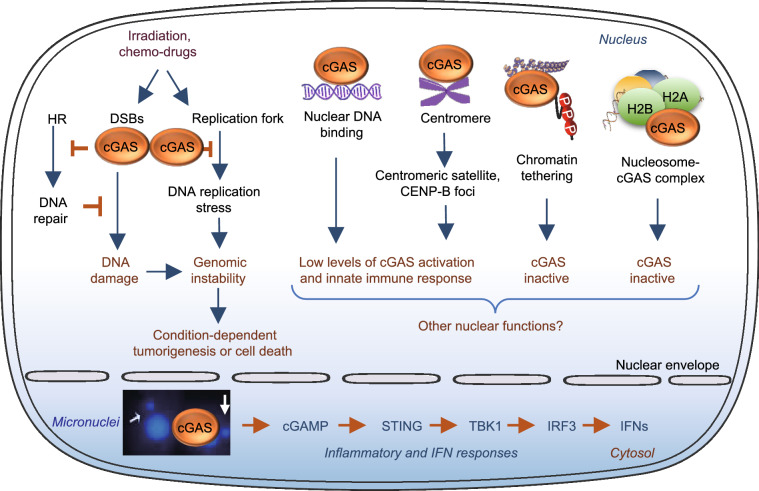
**Nuclear function of cGAS.** Irradiation or chemo-drugs induces DNA damage, leading to increased micronuclei and genomic instability. In the DNA damaged nucleus, cGAS binds to the double-stranded DNA breaks (DSBs), where it inhibits homologous recombination (HR)-mediated DNA repair, leading to uncontrolled genomic stability and tumorigenesis. On the other hand, cGAS could suppress DNA replication stress-induced DNA damage by slowing replication forks through a direct binding of DNA replication components proliferation cell nuclear antigen (PCNA), which leads to a resistance to radiation and chemotherapy in cancer cells. Increasing cGAS nuclear translocation and its binding to centromere at centromeric satellite or CENP-B foci are associated with low levels of cGAS activation and innate immune response. On the other hand, the binding to nucleosomes, chromatin tethering, and N-terminal hyperphosphorylation is associated with cGAS inactivation. Whether the nuclear localized cGAS has additional function remains to be further determined

The finding that cGAS is localized in the nucleus raises a very interesting question as to whether nuclear cGAS has functions beyond that of cytosolic DNA sensing, especially with the finding that the binding to chromatin or nucleosome greatly abolishes the enzymatic activity and innate immune response of cGAS. Here we summarize several recent findings on the potential roles of cGAS in the nucleus, including suppression of homologous-recombination-mediated DNA repair and promotion of tumorigenesis (Liu et al., 2018; Jiang et al., 2019), attenuation of DNA replication (Chen et al., 2020), and regulation of innate immune response (Gentili et al., 2019; Cui et al., 2020) (Fig. 4).

### Nuclear cGAS inhibits DNA repair and promotes tumorigenesis via a STING-independent mechanism

Homologous recombination (HR) accurately repairs DSBs, which provides an efficient mechanism to preserve genome integrity. DNA damage has been found to induce cGAS nuclear translocation and its recruitment to the DSBs, where cGAS suppresses HR-mediated DNA repair (Liu et al., 2018) (Fig. 4). The inhibition of HR by cGAS depends on cGAS nuclear localization since retention of cGAS in the cytosol by replacing the B-lymphoid tyrosine kinase (BLK) phosphorylation site Tyr^215^ with a glutamate in cGAS or knocking down BLK abolishes the inhibitory effect of cGAS on HR. The inhibition of HR-induced DNA repair is independent on the enzyme activity and DNA binding property of cGAS, since mutating key residues in either the DNA-binding site or the enzyme active site of cGAS had no effect on cGAS-mediated inhibition of HR-induced DNA repair. In the nucleus, DNA damage induces the interaction of cGAS with the phosphorylated H2A histone family member X (γH2AX) (Liu et al., 2018), which is a well-characterized marker of DNA DSBs (Rogakou et al., 1998). The interaction is mediated by the C-terminal region of cGAS (amino acids 213–522) and the phospho-epitope of γH2AX (Ser^139^). γH2AX recruits cGAS to the DNA-damage sites and facilitates its interaction with Poly (ADP-ribose) polymerase 1 (PARP1), which impedes the formation of the PARP1-Timeless complex and thereby suppresses HR (Liu et al., 2018). At DSB, cGAS suppresses exclusively on HR-mediated repair, but leaving the error prone non-homologous end joining (NHEJ) pathway unaffected (Liu et al., 2018). Knockdown of cGAS suppresses DNA damage and inhibits the growth of Lewis lung carcinoma (LLC) cells both *in vitro* and *in vivo*, uncovering a tumor-promoting role of nuclear cGAS. The finding that cGAS modulates HR is further supported by the finding that chromatin-bound cGAS inhibits HR-directed DNA repair by compacting of bound template dsDNA into a higher-ordered state that is less amenable to strand invasion by DNA repair protein RAD51-coated single-stranded DNA filaments in a STING-independent manner (Jiang et al., 2019). Taken together, these findings reveal that cGAS has a promoting effect on tumor growth by inhibiting HR-mediated DNA repair, revealing an innate immune-independent function of cGAS. Thus, targeting nuclear cGAS may be a useful approach for cancer prevention and therapy (Liu et al., 2018; Jiang et al., 2019).

### Nuclear cGAS binds to replication forks and attenuates DNA replication

cGAS has been found to inhibit cell proliferation by binding to nuclear DNA during S phase of the cell cycle (Chen et al., 2020). Both wild-type and the cGAMP synthase inactive cGAS rescued fast proliferation of cGAS-deficient cells, suggesting that cGAS slows cell proliferation in a cGAMP-independent manner. RNA sequencing (RNA-seq) analysis revealed that the genes involved in DNA replication and the cell cycle are significantly up-regulated by the loss of cGAS in human foreskin fibroblasts (BJ cells). In the nucleus, cGAS interacts with replication fork proteins in a DNA binding-dependent manner. The binding of cGAS to DNA slows replication forks via a STING-independent mechanism and cGAS deficiency accelerates fork replication but compromises fork stability, leading to increased sensitive to radiation and chemotherapy in U2OS cells, a human osteosarcoma epithelial cell line (Chen et al., 2020) (Fig. 4). These findings suggest that by slowing DNA replication forks, cGAS could suppress replication-associated DNA damage and could thus be an attractive anti-tumor therapeutic target.

### The role of nuclear localized cGAS in innate immune response

While many studies show that the enzyme activity of cGAS is suppressed in the nucleus by binding to nucleosomes (Boyer et al., 2020; Kujirai et al., 2020; Michalski et al., 2020; Pathare et al., 2020; Zhao et al., 2020) or tethering with chromatin (Volkman et al., 2019), Gentili et al. (2019) found that nuclear localized cGAS, which is associated with centromeres and the long interspersed nuclear element (LINE) DNA repeats, is able to synthesize cGAMP and induce innate immune activation in primary human monocyte-derived dendritic cells (DCs). The nuclear localization and centromere association of cGAS are regulated by the N-terminal domain of the protein and the activity of nuclear cGAS is 200-fold less active toward self-DNA than exogenous cytosolic DNA (Gentili et al., 2019). In the nucleus, cGAS was also found to be activated by human immunodeficiency virus (HIV) through interacting with NONO (non-POU domain-containing octamer-binding protein) in DCs and macrophages (Lahaye et al., 2018). Interestingly, the nuclear-localized cGAS has been shown to activate the innate immune response through a DNA sensing-independent mechanism in various cells under RNA virus infection (Cui et al., 2020). In these cells, the nuclear-localized cGAS interacts with the protein arginine methyltransferase 5 (PRMT5), which catalyzes the symmetric demethylation of histone H3 arginine 2 at interferon beta (*Ifnb*) and interferon alpha 4 (*Ifna4*) promoters, thus facilitating the access of IRF3 to promote IFN production. This study uncovers a novel mechanism by which the nuclear localized cGAS promotes *Ifn* gene expression and the innate immune response.

### Mechanisms regulating cGAS nuclear translocation

**Figure 5:**
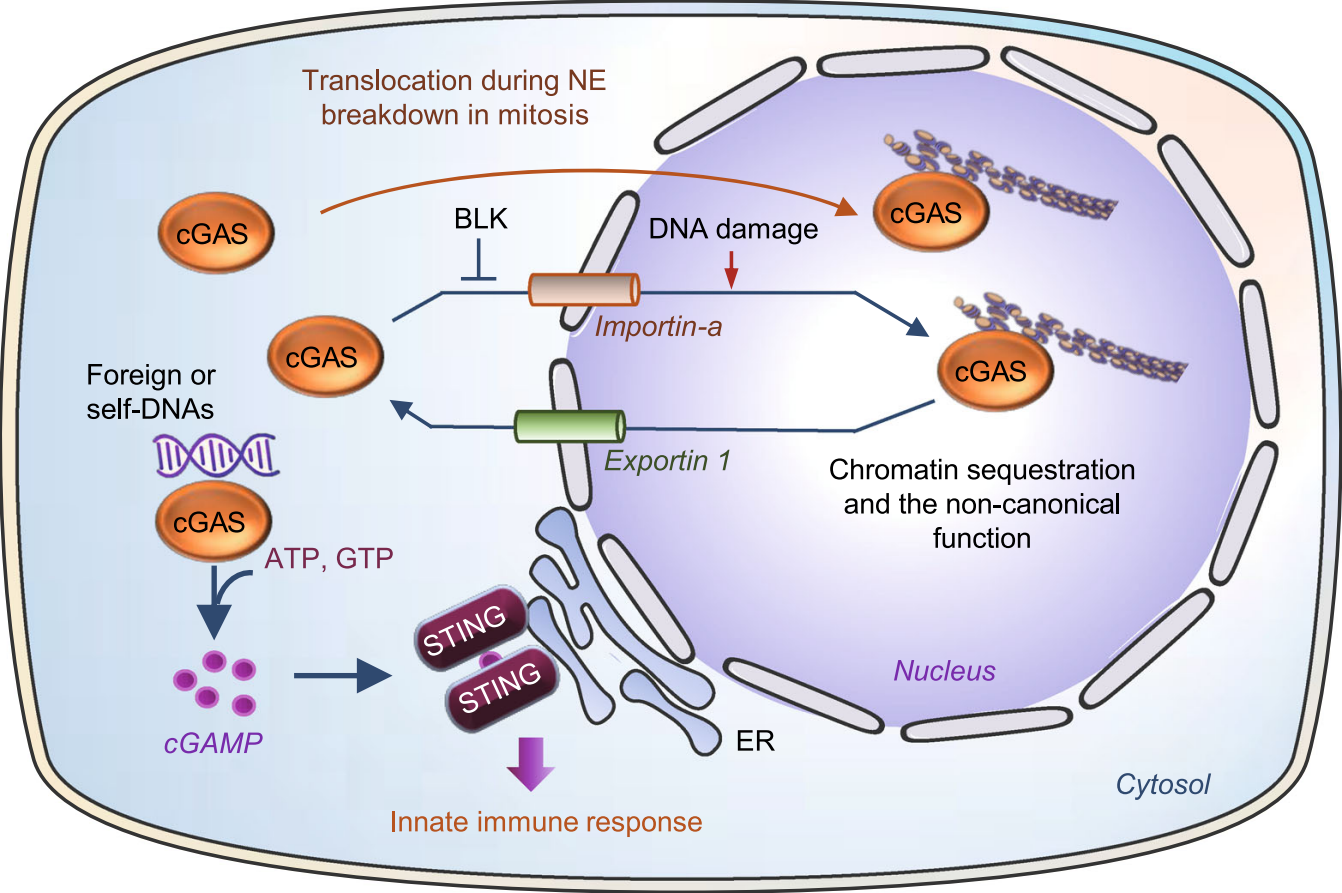
**Regulation of cGAS nuclear translocation**. Cytosolic cGAS may translocate into the nucleus via an importin α-dependent mechanism, which is stimulated by DNA damage but inhibited by BLK-mediated phosphorylation. cGAS could also translocated to the nucleus during mitosis when the nuclear envelop (NE) is broken. Nuclear cGAS could also be exported to cytosol through Exportin 1. The translocation mechanism of cGAS is related to its distinct function in cellular sublocation

Whereas it is well established that cGAS undergoes nuclear translocation, the precise mechanism regulating the translocation process remains elusive. It has been suggested that endogenous nuclear cGAS in interphase could result from the interaction with nuclear DNA during nuclear envelope (NE) breakdown that occurred in a previous mitosis (Mackenzie et al., 2017; Gentili et al., 2019). Indeed, cGAS is found to be associated with nuclear chromatin DNA during mitosis in proliferating cells (Yang et al., 2017; Gentili et al., 2019; Zhong et al., 2020). However, it has been found that cGAS nuclear translocation could also be promoted by DNA damage even the NE was intact (Raab et al., 2016; Liu et al., 2018). Two nuclear localization sequences, NLS1 and NLS2, have been identified in cGAS (Liu et al., 2018), suggesting a potential classic importin-dependent nuclear translocation mechanism. In response to DNA damage, both NLS2 and suppression of B-lymphoid tyrosine kinase (LTK)-mediated phosphorylation at Tyr^215^ of cGAS are required for its nuclear translocation, which is uncoupled from the function in DNA sensing (Liu et al., 2018). In addition to NLSs, a nuclear export signal (NES) has also been identified in cGAS (Sun et al., 2021). Mutation of NES and blocking nuclear exporting function of CRM1 (the chromosomal maintenance 1, also known as Exportin 1) by leptomycin B greatly increased the sequestration of cGAS within the nucleus and the loss of cytosolic DNA-induced interferon response (Sun et al., 2021) (Fig. 5). Multiple phosphorylation sites have been identified in cGAS during mitosis (Zhong et al., 2020; Li et al., 2021), but whether phosphorylation at these sites plays a role in cGAS nuclear translocation has yet to be determined.

## SURVEILLANCE OF MICRONUCLEI BY cGAS LINKS GENOME INSTABILITY TO INNATE IMMUNITY

In addition to sensing cytosolic DNA to initiate the innate immune defense, cGAS has also been found to play a critical part in the surveillance of micronuclei, an extra-nuclear body that contains whole or partial chromosomes failed to be included in the main nucleus after mitosis (Harding et al., 2017; Mackenzie et al., 2017). Micronuclei could be induced by genotoxic stress and chromosomal instability (CIN) (Fenech et al., 2011), the latter is characterized by persisting errors in chromosome segregation during mitosis, which is critical in promoting tumorigenesis (Lengauer et al., 1998). Some recent studies show that CIN is also involved in the crosstalk between a tumor and its surrounding microenvironment, which is mediated by the cGAS-STING pathway (Bakhoum et al., 2018; Hong et al., 2019; Nassour et al., 2019). cGAS recognizes dsDNA in micronuclei and the localization of cGAS to micronuclei is induced by genome instability in a mouse model of monogenic autoinflammation and in human cancer cells with exogenous DNA damage (Mackenzie et al., 2017). When micronuclei rupture, DNA becomes accessible for cGAS binding, leading to the activation of the cGAS-STING pathway and consequent increase in type I IFN response (Mackenzie et al., 2017) (Fig. 4). Thus, recognition of micronuclei by cGAS may act as a cell-intrinsic immune surveillance mechanism that detects a range of neoplasia-inducing processes. One study reported that micronuclei-localized cGAS is originated from nucleus-bound cGAS, indicating the membrane collapse may not even be necessary for cGAS activation by micronuclei (Jiang et al., 2019). Bakhoum et al. (2018) found that in CIN-high breast cancer cells, higher chromosome segregation error-induced cytosolic micronuclei trigger cGAS-STING perinuclear localization and the activation of the noncanonical NF-κB signaling pathways, which is associated with cancer cell migration and invasion (Bakhoum et al., 2018). In addition to genotoxic stress and CIN, the formation of micronuclei is also induced by DSBs which is associated with localization of cGAS in the micronuclei (Harding et al., 2017; Jiang et al., 2019). This DSBs-induced cGAS-STING activation and inflammatory response is dependent on cell cycle progression through mitosis, which is an important mechanism to explain the genotoxic cancer therapy-induced delayed on sets inflammatory response in contrast to the acute DNA-damage response. cGAS also senses cytosolic chromatin fragments (CCFs), a common feature of senescent cells, and knockdown of the NE protein lamin B1 or loss of NE integrity by irradiation triggers the activation of cGAS pathway (Gluck et al., 2017). Activation of the cGAS-STING pathway is also induced by cytosolic telomeric DNA, micronuclei, CCFs and nucleoplasmic DNA bridges, in replicative crisis cells, which plays a key role in promoting autophagic cell death and suppressing tumorigenesis (Nassour et al., 2019). Replicative crisis is a senescence-independent process that functions as the final barrier to prevent cells from evolving towards malignancy. While most of cells die during crisis, occasionally escaped cells with characteristics of malignant transformation may lead to tumorigenesis (Artandi and DePinho, 2000). Nassour et al. (2019) found that during replicative crisis in which the NE is fragile and undergo spontaneous disruption, telomeric DNA damage promotes cytosolic chromatin fragmentation that activates the cGAS-STING pathway, triggering macroautophgy. Inhibition of autophagy prevents crisis and promotes CIN (Nassour et al., 2019), which is consistent with the previous finding that autophagy prevents CIN and associated tumorigenesis (Karantza-Wadsworth et al., 2007; Mathew et al., 2007). These findings suggest that the cGAS-STING pathway functions as a tumor suppressor by inducing autophagy, which plays a critical role in the elimination of cells in crisis. Interestingly, cGAS has recently been found to act as a micronucleophagy receptor involved in the clearance of micronuclei via interaction with MAP1LC3B (microtubule associated protein 1 light chain 3B) in the autophagic machinery (Zhao et al., 2021). In contrast to its DNA sensing function to activate micronuclei-driven inflammation, cGAS-mediated micronucleophagy reduces genotoxic stress-induced cGAMP production, uncovering an unprecedented role of cGAS in reducing innate immune surveillance (Zhao et al., 2021).

## INHIBITION OF cGAS ACTIVITY BY PHOSPHORYLATION AND CHROMATIN TETHERING DURING MITOSIS

**Figure 6:**
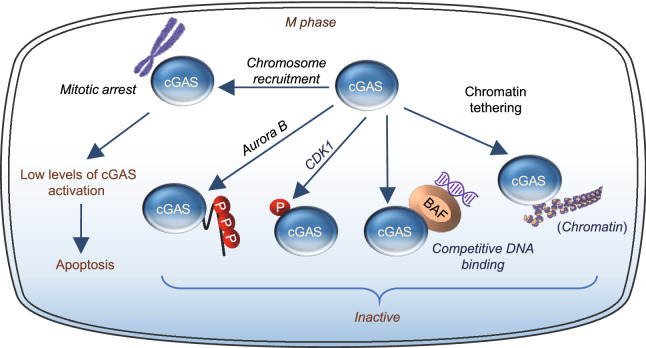
**cGAS functions during mitosis.** During mitotic arrest, cGAS is recruited to mitotic chromosomes, where it can be activated at a low level to promotes apoptosis. During normal mitosis process, cGAS activity is inhibited by several safeguard mechanisms including mitotic kinase Aurora B- or CDK1-medicated phosphorylation, chromatin tethering and BAF competitive DNA binding

A unique feature of the cell cycle is the disassembly and reassembly of the nucleus. At the beginning of mitosis, the chromosomes condense and the NE breaks down, resulting in most of the nuclear contents exposed into the cytoplasm. While cGAS is known to associate with chromatin after NE breaking down, it is not activated by DNA at the mitotic phase of the cell cycle (Guey et al., 2020; Zhong et al., 2020; Li et al., 2021), suggesting the presence of a mechanism(s) to suppress cGAS activity at this phase. Consistent with this, human cGAS is phosphorylated by the mitotic cyclin-dependent kinase 1 (CDK1)-cyclin B complex at a highly conserved site Ser^305^ (Zhong et al., 2020). Phosphorylation at this site inhibits the ability of cGAS to synthesize cGAMP upon mitotic entry, leading to unresponsiveness to DNA-triggered innate immunity in mitotic cells (Zhong et al., 2020) (Fig. 6). Human cGAS is also phosphorylated by the mitotic kinase Aurora kinase B at multiple sites of the N-terminus during mitosis (Li et al., 2021), which suppresses cGAS activity by blocking its DNA binding and liquid phase separation (Li et al., 2021). By a series of elegant cellular and biochemical studies, Li et al. (2021) showed that the N-terminus is critical for cGAS to sense chromatin DNA but not mitochondrial DNA. In addition to the in-cis phosphorylation-mediated mechanism that inactivates cGAS, cGAS activity is also suppressed by chromatin tethering that acts in trans to prevent cGAS oligomerization and thus activation during mitosis. The DNA binding of cGAS was also competitively suppressed by the barrier-to-autointegration factor (BAF), a chromatin-binding protein that is essential for nuclear membrane reformation at the end of mitosis (Guey et al., 2020). BAF-deficient cells showed repetitive NE rupture events, which were strongly corelated with the accumulation of cGAS within discrete in trinuclear foci and a robust interferon gene signature (IGS) response. Thus, BAF acts as a sentinel to restrict the binding of cGAS to DNA and thus activation. Golgi vesiculation during mitosis restricts STING activation also provides second barrier to prevent cGAS-induced innate immunity (Uhlorn et al., 2020) (Fig. 6). Together, all these findings suggest the presence of tightly regulated safeguard mechanisms that prevent cGAS from abnormal activation by chromatin DNA during the cell cycle, thus ensuring normal cell proliferation, division, and genome stability.

While the activity of cGAS is suppressed during mitosis, a non-canonical role of cGAS was also reported as an instigator of mitotic cell death during prolonged mitotic arrest, independent to its innate immune function (Zierhut et al., 2019). cGAS is recruited to mitotic chromosomes and this localization was not affected by mutations in the cGAS DNA binding domain (Zierhut et al., 2019). When cells arrest in mitosis, cGAS activation promotes cell death by suppressing Bcl-xL-dependent inhibition of mitochondrial outer membrane permeabilization (MOMP). The apoptotic-promoting effect of cGAS during mitotic arrest is dependent on low levels of IRF3 phosphorylation but not IFN production. The effect of cGAS on apoptosis appears to be selective, because cGAS depletion had no effect on timing of UV irradiation-induced cell death (12). These findings suggest that the cGAS pathway may operate in two distinct modules depending on the transcriptional competence and apoptotic potential of the cell (Zierhut et al., 2019) (Fig. 6). In transcriptionally competent interphase cells, cGAS promotes inflammation but has low apoptotic potential. In transcriptionally attenuated mitotic cells, the cGAS signaling has high apoptotic potential and is able to induce apoptosis via an IRF3 phosphorylation-dependent but IFN production-independent mechanism.

## CONCLUDING REMARKS AND PERSPECTIVES

Over the past decade, our understanding on the role of cGAS in host defense has advanced considerably (Ablasser and Chen, 2019; Motwani et al., 2019). The ability of cytosolic cGAS to recognize self- and non-self-DNAs and to couple this recognition to innate immune responses has emerged as an important mechanism for the development of pharmacological drugs to treat chronic inflammation and cancer. However, numerous studies have found that cGAS is also localized in the nucleus and exerts STING-independent functions (Orzalli et al., 2015; Xia et al., 2016; Liu et al., 2018; Gentili et al., 2019; Jiang et al., 2019; Volkman et al., 2019; Zierhut et al., 2019; Michalski et al., 2020; Zhao et al., 2020), suggesting more broad roles of cGAS in addition to functioning as a DNA sensor.

A dogma on the prevention of cGAS from activation by self-DNAs such as mtDNAs and nuclear DNAs is the compartmentalization of these molecules in distinct locations in the cell (Gekara and Jiang, 2019). Indeed, cGAS is found to be localized at the plasma membrane in macrophages and this localization has been suggested as a mechanism that prevents cGAS from activation by self-DNA in the cytosol (Barnett et al., 2019). The biological significance of cGAS nuclear localization, however, remains somewhat ambiguous, given that most cellular self-DNA is located in the nucleus. cGAS is found to be associated with distinct nuclear sub-structures such as nucleosomes (Kujirai et al., 2020; Pathare et al., 2020), replication forks (Chen et al., 2020), DSBs (Liu et al., 2018), and centromeres and LINE DNA repeats (Gentili et al., 2019). A number of mechanisms such as chromatin tethering, hyperphosphorylation, DNA binding blocking, and dimerization blunting have been implicated in inactivating nuclear or chromatin-bound cGAS, but whether nuclear cGAS has a cGAMP enzymatic-independent function remains to be further explored. For example, cGAS interacts with nucleosome by binding with the acidic patch of the heterodimer histone H2A-H2B (Boyer et al., 2020; Kujirai et al., 2020; Michalski et al., 2020; Pathare et al., 2020; Zhao et al., 2020), which are important proteins involved in the regulation of chromatin package, remodeling, stability, heterochromatin compaction, DNA damage and repair, genome epigenetic regulation, and gene activation (Hauer and Gasser, 2017; Martire and Banaszynski, 2020). An interesting question remains to be answered is whether cGAS plays any role in regulating those cellular processes and whether the binding of cGAS to histones is dynamically regulated. Another puzzle remains to be solved is how cGAS is activated by chromatin fragments in micronuclei but not by the abundant nuclear chromatin in the nucleus. Is the activation of cGAS in micronuclei by damaged chromosomal DNA in micronuclear chromatin, or by some uncharacterized factors present in micronuclei but not in the nucleus? Future investigations should shed light on these possibilities.

cGAS is known to interact with chromatin during mitosis (Yang et al., 2017; Zierhut et al., 2019; Li et al., 2021), but the precise mechanism and the role of cGAS in mitosis remain unclear. To allow faithfully segregation of chromosomes during mitosis, the homogenously distributed chromatin need to be extensively compacted to form highly condensed chromosomes by histone (Wilkins et al., 2014; Antonin and Neumann, 2016). This finding raises a question as to how precisely cGAS is localized at this phase. Gentili et al. (2019) find that nuclear cGAS is enriched at the foci of centromere protein B (CENP-B), a protein that plays a critical role in the assembly of specific centromere structures in interphase nuclei and on mitotic chromosomes. This finding suggests a possible functional association of nuclear cGAS with centromeres. However, it is current unknown whether nuclear cGAS plays a role in regulating kinetochore assembly and chromosome segregation, a major function of the centromere. In addition, cGAS is phosphorylated by the major mitotic kinase CDK1-cyclin B complex (Zhong et al., 2020) and by Aurora kinase B (Li et al., 2021), kinases that play key roles in the regulation of mitosis phase transition and chromosomal segregation. Although hyperphosphorylation of cGAS at its N-terminal and tethering with chromatin greatly inhibit its enzymatic function, whether cGAS plays a role in regulating mitotic cellular event remains unknown. Further investigations will be needed to elucidate the potential roles and the mechanisms of action of nuclear cGAS in the regulation of key cellular processes such as cell cycle, cell division, epigenetic regulation, senescence and beyond.

The DNA-repair system provides a mechanism to cope with the frequent environmental insults that may compromise the genomic integrity of the organism and predispose the organism to immunodeficiency and cancer (Hakem, 2008). An important finding on the functional role of nuclear cGAS is that the nuclear localized molecule may promote tumorigenesis by inhibiting DNA repair, leading to genome instability (Liu et al., 2018; Jiang et al., 2019; Chen et al., 2020). Thus, targeting nuclear cGAS may be a useful approach for cancer prevention and therapy. However, it is interesting to note that under certain conditions such as genotoxic stress and CIN, cGAS may also function as a tumor suppressor by acting as an innate immune surveillant that detect various neoplasia-inducing processes (Mackenzie et al., 2017; Zhao et al., 2021). Indeed, cGAS in the cytosol has been shown to play a critical role in the surveillance and clearance of micronuclei (Harding et al., 2017; Mackenzie et al., 2017; Bakhoum et al., 2018). In addition, activation of the cGAS-STING pathway suppresses tumorigenesis by inducing autophagic cell death during replicative crisis (Nassour et al., 2019). Therefore, cGAS may act as a double-edged sword in the progress of tumorigenesis. More knowledge on the cytosolic and nuclear function of cGAS and its dynamic regulation would be important in developing specific cGAS-based anti-cancer therapies.

Lastly, while cytosolic and nuclear localization of cGAS has been found in many cells (Orzalli et al., 2015; Mackenzie et al., 2017; Lahaye et al., 2018; Liu et al., 2018; Gentili et al., 2019; Volkman et al., 2019; Zierhut et al., 2019; Chen et al., 2020; Cui et al., 2020; Michalski et al., 2020; Pathare et al., 2020; Zhao et al., 2020), there is an evidence showing that cGAS is localized predominantly at the plasma membrane in macrophages (Barnett et al., 2019). The cause for this controversy is currently unknown, but different experimental conditions may provide some explanation (Barnett et al., 2019; Volkman et al., 2019). However, it is also entirely possible that the distinct subcellular localization of cGAS is spatiotemporally regulated and cell type-dependent, which may be essential for the unique functions of this protein. Additional work to uncover the functions of the nuclear, plasma membrane and cytosolic localized cGAS and the signaling cues that control cGAS sub-cellular localization should improve our understanding of cGAS function in DNA sensing and beyond.
